# Eribulin improved the overall survival from the initiation of first-line chemotherapy for HER2-negative advanced breast cancer: a multicenter retrospective study

**DOI:** 10.1186/s12885-021-09137-0

**Published:** 2022-01-03

**Authors:** Shogo Nakamoto, Junichiro Watanabe, Shoichiro Ohtani, Satoshi Morita, Masahiko Ikeda

**Affiliations:** 1 Division of Breast Oncology, Shizuoka Cancer Center, 1007 Shimonagakubo, Nagaizumi, Shizuoka, 411-8777 Japan; 2 Division of Breast and Thyroid Gland Surgery, Fukuyama City Hospital, 5-23-1 Zao, Fukuyama, Hiroshima, 721-8511 Japan; 3 Division of Breast Surgery, Hiroshima City Hiroshima Citizens Hospital, 7-33 Motomachi, Naka-ku, Hiroshima, Hiroshima 730-8518 Japan; 4 Division of Biomedical Statistics and Bioinformatics, Kyoto University Graduate School of Medicine, Yoshida-Konoe, Sakyo-ku, Kyoto, 606-8501 Japan

**Keywords:** Advanced breast cancer, Eribulin, HER2-negative, Overall survival, Real world

## Abstract

**Background:**

Eribulin methylate (eribulin) improved the overall survival (OS) of eribulin-treated patients with HER2-negative advanced breast cancer (ABC) in prospective and retrospective studies. However, the effect of eribulin on OS as first-line chemotherapy and the characteristics of the patients who benefited from eribulin remain unclear.

**Methods:**

Between January 2011 and December 2016, 301 patients with HER2-negative ABC who started first-line chemotherapy at 3 institutions were retrospectively evaluated for OS from the initiation of first-line chemotherapy.

**Results:**

We identified 172 patients (119 estrogen receptor-positive [ER+], 47 ER−, 6 unknown) who received eribulin (eribulin group) and 129 patients (92 ER+, 31 ER−, 6 unknown) who did not receive eribulin (non-eribulin group). The median OS from the initiation of first-line chemotherapy in the two groups was not statistically significant (869 vs. 744 days, *P* = 0.47, log-rank); however, in patients who received eribulin in later lines (≥3rd-line) and who had a history of perioperative chemotherapy with anthracycline- and/or taxane-based regimens, the median OS improved (1001 vs. 744 days, *P* = 0.037; and 834 vs. 464 days, respectively *P* = 0.032, respectively; Wilcoxon). Multivariate analyses revealed that a history of perioperative chemotherapy with anthracycline- and/or taxane-based regimens was a predictive factor (hazard ratio, 0.39; 95% confidence interval, 0.21–0.70) for OS.

**Conclusions:**

This study successfully identified subgroups of HER2− ABC patients with improved OS by eribulin therapy. Selecting patients according to their background and line of treatment will maximize the efficacy of eribulin therapy.

## Background

Eribulin methylate (eribulin) is a novel antitubulin agent widely used for patients with human epidermal growth factor receptor-2 negative (HER2−) advanced breast cancer (ABC). It is a preferred treatment option for patients with a history of anthracycline- and/or taxane-based therapy [1, 2]. In the EMBRACE study, the efficacy of eribulin was compared with treatment of the physician's choice (TPC) in patients with heavily pretreated HER2− ABC. Although there was no statistically significant difference in progression-free survival (PFS), overall survival (OS) was significantly improved in patients treated with eribulin compared with those receiving TPC treatment (hazard ratio [HR], 0.81; 95% confidence interval [CI], 0.66–0.99; *P* = 0.041, log-rank) [3]. In study 301, which included HER2− ABC patients who had ≤1 prior regimen for ABC, eribulin showed a trend of improved OS compared with capecitabine; however, there was no statistically significant difference (HR, 0.88; 95% CI, 0.77–1.00; *P* = 0.056, log-rank) [4]. A pooled analysis of these phase 3 studies demonstrated a significant OS benefit (HR, 0.85; 95% CI 0.77–0.95; *P* = 0.003, log-rank) of eribulin compared with controls [5], and another pooled analysis of patients who received at least one prior chemotherapy, extracted from the same dataset, showed a significantly superior OS (HR, 0.85; 95% CI, 0.76–0.94, *P* = 0.002) in the eribulin group compared with the controls [6]. Although several reports have alluded to an improvement in OS following eribulin therapy compared with conventional chemotherapy in ABC patients in real-world settings [7–9], the background of the patients likely to experience the most benefit from eribulin therapy has not been established.

In addition, most previous studies that report a prolongation of OS by eribulin therapy discuss the improvement in OS from the initiation of eribulin therapy. Therefore, whether or not eribulin therapy at any treatment line improves OS from the initiation of first-line chemotherapy remains unclear.

Therefore, we retrospectively examined real-world data of HER2− ABC patients from three institutions to evaluate the effect of eribulin therapy on OS from the initiation of first-line chemotherapy and to identify the subgroup of patients who are likely to receive an OS benefit from eribulin therapy.

## Methods

### Patients

We retrospectively evaluated HER2− ABC patients who had started first-line chemotherapy at three registered sites (Fukuyama City Hospital, Hiroshima City Hiroshima Citizens Hospital, and Shizuoka Cancer Center) between January 2011 and December 2016. The dataset was identical to that from our previous report [10]. Physicians extracted the medical information of the patients who were treated at these institutions from the medical records [10]. We did not use specific case-report forms. The medical information included patient characteristics and the data related to treatment efficacy. Treatment response was assessed according to the Response Evaluation Criteria in Solid Tumors (RECIST) version 1.1 [11]. The surveillance interval was defined by each physician’s judgment based on individual patient need.

### Treatments

First-line and subsequent chemotherapeutic regimens were determined based on the physician's judgment and/or patient preferences. Dose modification and interruption or discontinuation of chemotherapy was done by the physician decision based on the patient’s condition.

The chemotherapeutic regimens used other than eribulin were as follows: anthracycline-based (such as epirubicin + cyclophosphamide), taxane monotherapy, paclitaxel + bevacizumab, 5-fluorouracil derivatives (such as capecitabine, S-1 [combination drug of Tegafur, Gimeracil and Oteracil Potassium]), and “others” (e.g., vinorelbine, gemcitabine). In Japan, eribulin is approved for use and reimbursed when administered at any line of chemotherapy, so first-line-use is available for ABC patients.

### Statistical analyses

Before performing survival analyses, patients were divided into two subgroups (eribulin and non-eribulin) according to the therapy received during the observational period. Patients who had received eribulin were classified into the eribulin group, whereas those who had never received eribulin were classified into the non-eribulin group. We defined OS as the duration from the initiation of first-line chemotherapy to death from any cause.

A Wilcoxon’s rank sum test was used to compare the median age and Fisher’s exact test was used to compare the proportions of categorical variables between groups. Survival analyses were estimated using the Kaplan–Meier method and comparisons between groups were made using the log-rank test or the generalized Wilcoxon test. For univariate and multivariate analyses, we used Cox regression models. A *P* value of <0.05 was considered statistically significant. The analyses were performed using the EZR software program (Saitama Medical Center, Jichi Medical University, Saitama, Japan), which is a graphical user interface for the R software program (The R Foundation for Statistical Computing, Vienna, Austria) [12].

## Results

### Patient characteristics

We evaluated 301 HER2− ABC patients treated at 3 institutions with 172 patients (119 estrogen receptor-positive [ER+], 47 ER−, 6 unknown) receiving eribulin (eribulin group) and 129 patients (92 ER+, 31 ER−, 6 unknown) not receiving eribulin (non-eribulin group). The median follow-up period was 21.9 months (range 0–77.3 months). The baseline patient characteristics at the initiation of first-line chemotherapy are shown in Table 1. The eribulin group included more patients with recurrent disease (74.4% vs. 60.5%, *P* = 0.012) and a history of perioperative anthracycline- and/or taxane-based therapy (54.7% vs. 41.1%, *P* = 0.020) compared with the non-eribulin group; however, no other significant differences were found.

**Table 1:** Patient characteristics at the time of the administration of first-line chemotherapy

	Eribulin, *n*	%	Non-Eribulin, *n*	%	*P* value
Total	172		129		
Median age, years (range)	58 (28-87)		60 (29-90)		0.29^a^
≥ 60 years	90	52.3	65	50.4	0.82
Estrogen receptor status					
Positive	119	69.2	92	71.3	0.59^b^
Negative	47	27.3	31	24.0	
Unknown	6	3.5	6	4.7	
Diagnosis					
Advanced	44	25.6	51	39.5	0.012
Recurrence	128	74.4	78	60.5	
Metastases					
Central nervous system	7	4.1	10	7.8	0.21
Bone	99	57.6	77	59.7	0.72
Lung	71	41.3	41	31.8	0.12
Pleura/ lymphangiopathy	37	21.5	30	23.3	0.78
Lymph node	114	66.3	92	71.3	0.38
Liver	69	40.1	41	31.8	0.15
Type of metastases					
Visceral	117	68.0	77	59.7	0.15
Non-visceral	55	32.0	52	40.3	
Number of metastatic sites					
≥ 3	99	57.6	76	58.9	0.91
< 3	73	42.4	53	41.1	
Perioperative chemotherapy					
Yes	94	54.7	53	41.1	0.020
No	78	45.3	76	58.9	
Disease-free interval					
< 24 months	92	53.5	82	63.6	0.099
≥ 24 months	80	46.5	47	36.4	
Eribulin treatment line					
≥ 3	84	48.8			
< 3	88	51.2			

^a^Wilcoxon’s rank sum test was performed.

^b^Comparing ER+ and ER-

^c^Treatment included anthracycline and/or taxane

### Overall survival

**Fig. 1:**
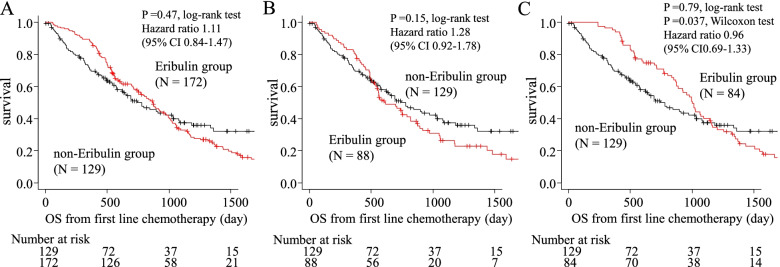
The overall survival in the eribulin and non-eribulin groups (**A**), eribulin group of patients who received eribulin at an early line (first or second) and non-eribulin group (**B**) and eribulin group of patients who received eribulin at a late line (third or later) and non-eribulin group (**C**). CI: confidence interval, OS: Overall survival

The median OS from the initiation of first-line chemotherapy did not significantly differ between the eribulin and non-eribulin groups (869 days vs. 744 days, HR = 1.11; 95% CI, 0.84–1.47, *P* = 0.47, log-rank; Fig. 1A), so we performed additional survival analyses based on the treatment line of eribulin. Eighty-eight of 172 patients in the eribulin group received eribulin during first or second line therapy, whereas 84 received eribulin at a later line (third or later). While early eribulin treatment resulted in no OS benefit compared with the non-eribulin group (median OS, 616 vs. 744 days; HR, 1.28 [95% CI, 0.92-1.78]; *P* = 0.15, log-rank; Fig. 1B), later line treatment showed a significant OS benefit according to a Wilcoxon’s test (median OS, 1001 vs. 744 days; HR, 0.96 [95% CI, 0.69–1.33]; *P* = 0.79, log-rank, *P* = 0.037, Wilcoxon; Fig. 1C) compared with the non-eribulin group. We performed univariate and multivariate analyses to identify independent factors influencing OS from the initiation of first-line chemotherapy (Table 2). In multivariate analyses, ER-negative status (HR 1.79; 95% CI: 1.29–2.48), bone metastases at the initiation of first-line chemotherapy (HR 1.51; 95% CI: 1.10–2.07), liver metastases at the initiation of first-line chemotherapy (HR 1.61; 95% CI: 1.14–2.28), a disease-free interval <24 months (HR 1.39; 95% CI: 1.04–1.87), and perioperative anthracycline- and/or taxane-based regimen (HR 1.66; 95% CI: 1.23–2.24) were all associated with poor OS, indicating that they are prognostic factors for HER2− ABC patients undergoing first-line chemotherapy.

**Table 2:** Univariate and multivariate analyses for the overall survival (Cox hazard model)

	Univariate			Multivariate		
	HR	95% CI	*P*	HR	95% CI	*P*
Age ≥ 60 years	1.23	0.94–1.62	0.13			
Estrogen receptor negative	1.53	1.14–2.07	0.005	1.79	1.29–2.48	<0.001
Recurrent disease	1.13	0.84-1.52	0.43			
Central nervous system metastasis	0.99	0.51–1.94	0.98			
Bone metastasis	1.40	1.06–1.85	0.018	1.51	1.10–2.07	0.011
Lung metastasis	1.04	0.78–1.37	0.81			
Pleura/lymphangiopathy metastasis	1.25	0.90–1.73	0.19			
Lymph node metastasis	1.07	0.80–1.43	0.66			
Liver metastasis	1.85	1.40-2.44	< 0.001	1.61	1.14-2.28	0.007
Visceral metastasis	1.54	1.15-2.07	0.004	1.22	0.83-1.80	0.31
≥ 3 metastatic sites	1.43	1.08–1.88	0.013	1.26	0.91–1.75	0.16
Perioperative chemotherapy ^a^	1.37	1.05-1.80	0.022	1.66	1.23-2.24	0.001
Disease-free interval<24 months	1.37	1.03–1.80	0.029	1.39	1.04–1.87	0.028
Therapy						
Eribulin vs. non-Eribulin	1.11	0.84-1.47	0.47	0.85	0.63-1.15	0.29
Early-line eribulin ^b^ vs. non-Eribulin	1.28	0.92-1.78	0.15			
Late-line eribulin ^c^ vs. non-Eribulin	0.96	0.69-1.33	0.79			

CI: confidence interval, HR: hazard ratio

^a^Treatment included anthracycline and/or taxane

^b^Early line includes first or second lines of therapy

^c^Late line includes third or later lines of therapy

### Additional analyses

**Fig. 2:**
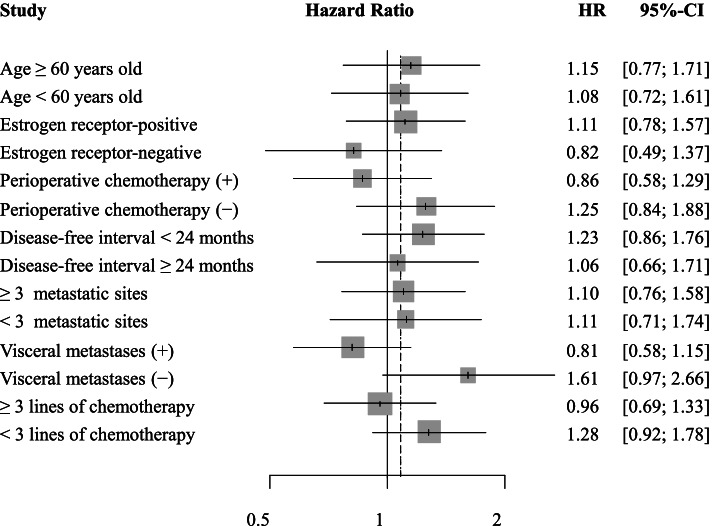
Hazard ratios for the overall survival. CI: confidence interval, HR: hazard ratio

We performed additional analyses using a Cox proportional hazard model to identify subgroups most likely to receive an OS benefit from eribulin therapy (Fig. 2); however, no independent factors for eribulin therapy were identified.

**Fig. 3:**
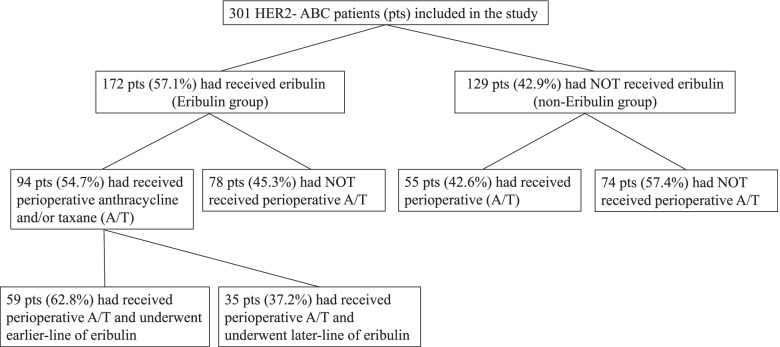
Breakdown of patients included in the study ABC: advanced breast cancer, A/T: anthracycline and/or taxane, HER2: human epidermal growth factor receptor-2 negative, pts: patients

**Fig. 4:**
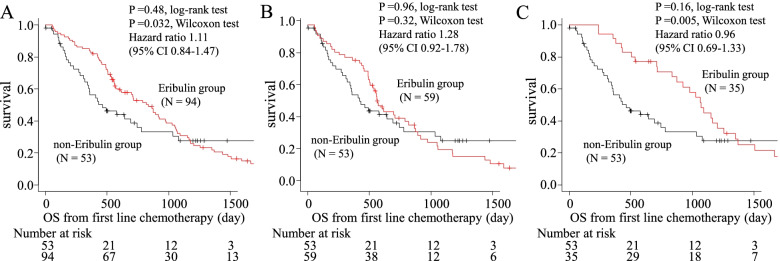
The subgroup of patients who had receive perioperative therapy with prior (neo) adjuvant anthracycline- and/or taxane-based regimens, the overall survival in the eribulin and non-eribulin groups (**A**), early-line eribulin group and non-eribulin group (**B**) and late-line eribulin group and non-eribulin group (**C**). OS: Overall survival

Eribulin has been approved in the United States and other countries for the treatment of ABC previously treated with at least two chemotherapeutic regimens, including anthracycline- and taxane-based regimens [1, 2]. Thus, we conducted additional analyses in the subgroup of patients who had received perioperative anthracycline- and/or taxane-based regimens (Fig. 3). The median OS was significantly longer in the eribulin group compared with the non-eribulin group according to a Wilcoxon’s test (834 days vs. 464 days, *P* = 0.48, log-rank, *P* = 0.032, Wilcoxon; Fig. 4A). In addition, we performed survival analyses on the treatment line of eribulin in the subgroup of patients who had received perioperative anthracycline- and/or taxane-based regimens. Fifty-nine out of 94 patients in the eribulin group received eribulin at an early line (first or second), whereas 35 had received it at a later line (third or later). Although early treatment did not result in an OS benefit compared with the non-eribulin group (median OS, 571 vs. 464 days, *P* = 0.96, log-rank *P* = 0.32, Wilcoxon; Fig. 4B), later treatment showed a significant OS benefit according to a Wilcoxon’s test compared with the non-eribulin group (median OS, 1070 vs. 464 days; HR, 0.96 [95% CI, 0.69–1.33]; *P* = 0.16, log-rank, *P* = 0.005, Wilcoxon; Fig. 4C).

We performed univariate and multivariate analyses to identify independent factors that influence OS (Table 3). In a multivariate analysis, ER-negative status (HR 1.99; 95% CI: 1.14–3.46), bone metastases at the initiation of first-line chemotherapy (HR 3.28; 95% CI: 1.84–5.82), a disease-free interval <24 months (HR 1.80; 95% CI: 1.04–3.10), visceral metastases at the initiation of first-line chemotherapy (HR 3.96; 95% CI: 1.94–8.08), and ≥3 metastases at the initiation of first-line chemotherapy (HR 0.55; 95% CI: 0.30–1.00) were associated with OS. Furthermore, eribulin therapy at a later line (third or later) was associated with a better OS compared with no eribulin therapy (HR 0.39; 95% CI: 0.21–0.70).

**Table 3:** Univariate and multivariate analyses of the overall survival in the subgroup of patients who had received perioperative therapy with prior (neo) adjuvant anthracycline- and/or taxane-based regimens (Cox hazard model)

	Univariate			Multivariate		
	HR	95% CI	*P*	HR	95% CI	*P*
Age ≥ 60 years	1.22	0.83–1.78	0.31			
Estrogen receptor negative	1.20	0.81–1.77	0.37	1.99	1.14–3.46	0.015
Central nervous system metastasis	1.20	0.53–2.74	0.66			
Bone metastasis	1.63	1.11–2.37	0.012	3.28	1.84–5.82	<0.001
Lung metastasis	1.11	0.76–1.63	0.59			
Pleura/lymphangiopathy metastasis	1.21	0.76–1.94	0.42			
Lymph node metastasis	1.10	0.75–1.62	0.63			
Liver metastasis	1.83	1.24-2.70	0.002	0.72	0.36-1.41	0.34
Visceral metastasis	1.74	1.14-2.63	0.009	3.96	1.94-8.08	<0.001
≥ 3 metastatic sites	1.39	0.95–2.01	0.087	0.55	0.30–1.00	0.050
Disease-free interval (< 24 months)	1.54	1.06–2.24	0.025	1.80	1.04-3.10	0.035
Therapy						
Eribulin vs. non-Eribulin	0.86	0.58-1.30	0.48			
Early line Eribulin ^a^ vs. non-Eribulin	1.01	0.65-1.57	0.96			
Late line Eribulin ^b^ vs. non-Eribulin	0.70	0.42-1.15	0.16	0.39	0.21-0.70	0.002

CI: confidence interval, HR: hazard ratio

^a^Early line includes first or second lines of therapy

^b^Late line includes third or later lines of therapy

## Discussion

As previously reported, OS from the initiation of first-line chemotherapy may be affected by the choice of subsequent therapy, increased tumor load, or a worsening performance status [16, 17]; thus, an improvement in OS from first-line chemotherapy with later-line chemotherapy appears challenging. A pivotal study of eribulin [3] and several real-life studies [8, 9] reported that eribulin improves OS after the initiation of eribulin; however, to our knowledge, no studies have evaluated OS from the initiation of first-line chemotherapy (including agents other than eribulin) or from the time of diagnosis of ABC, with the exception of one report based on a real-life setting [7]. In these circumstances, we successfully identified a subgroup with improved OS. Our multicenter, retrospective, observational study showed that the median OS from the initiation of first-line chemotherapy in the eribulin group was not significantly better than that in the non-eribulin group.

Perioperative anthracycline- and/or taxane-based regimens are commonly used for high-risk HER2− ABC patients [1, 2]; however, a certain number of patients will suffer cancer recurrence with anthracycline and/or taxane resistance. Under these circumstances, the optimal first-line chemotherapy for HER2− ABC patients who relapse after perioperative anthracycline and/or taxane therapy has been discussed. Two pivotal studies [13, 14] demonstrated improved OS following the initiation of first-line chemotherapy in patients with HER2− ABC patients who received perioperative anthracycline-based regimens; however, to our knowledge, no reports have documented a significant improvement in OS from the initiation of first-line chemotherapy for HER2− ABC that relapsed after perioperative anthracycline- and/or taxane-based therapy, in contrast to the findings in HER2-positive ABC patients [15].

Miller et al. [16] reported that paclitaxel plus bevacizumab as first-line chemotherapy for HER2− ABC improved median PFS (11.8 vs 5.9 months; P < 0.001, log-rank) compared with paclitaxel alone, and a subgroup analysis revealed that combination therapy resulted in a significant benefit, regardless of the perioperative chemotherapy regimen (none, anthracycline, or taxane). Although the experimental regimen resulted in a 40% reduction in the risk of disease progression (P < 0.001), OS did not significantly improve (median 26.7 vs 25.2 months; *P* = 0.16, log-rank). Discussions regarding the discrepancy between PFS and OS in the study have been made [17]; however, factors affecting OS, such as survival post-progression, crossover-use of drugs, or loss of follow-up, are more commonly encountered in real-world scenarios than in a clinical trial.

The efficacy of eribulin for HER2− ABC has been established in prospective reports. For example, in the EMBRACE study, eribulin treatment resulted in a significant improvement in median OS compared with TPC in patients with heavily pretreated ABC (13.1 months vs. 10.6 months, *P* = 0.041, log-lank) without a significant PFS improvement [3]. In addition, a pooled analysis of 2 prospective studies (EMBRACE and study 301) demonstrated a significant survival benefit of eribulin compared with the controls (15.2 months vs. 12.8 months HR 0.85; 95% CI: 0.77–0.95, *P* = 0.003, log-rank) [5]. Another pooled analysis based on the same dataset also revealed a significant superior OS in the eribulin group compared with the controls (15.0 months vs. 12.6 months HR 0.85; 95% CI: 0.76–0.94, *P* = 0.002, log-rank) [6]. Furthermore, eribulin improved the OS of patients with HER2− ABC not only in prospective studies [3–6], but also in retrospective studies [7–9]. According to a single-institutional retrospective study [7], eribulin therapy significantly improved OS from the diagnosis of ER+ HER2− ABC (HR, 0.67; 95% CI, 0.47–0.96; *P* = 0.025); however, the majority of real-world studies indicate an OS improvement from the initiation of eribulin therapy, not from the induction of first-line chemotherapy. A multi-institutional observation study using the Epidemiological Strategy and Medical Economics database showed that the median OS of HER2− ABC patients was significantly prolonged by late-line (e.g., third- and fourth-line) chemotherapy (eribulin therapy vs. other chemotherapeutic regimens: 11.27 vs. 7.65 months, P < 0.001; 10.91 vs. 5.95 months, P < 0.001) [8]. Kazmi et al. conducted a retrospective, observational study using data from the Cancer Treatment Centers of America to estimate OS in clinical practice of patients with ABC and visceral metastasis (liver or lung) treated in the third-line setting with eribulin, gemcitabine, or capecitabine. The results showed that patients receiving eribulin had a numerically higher median OS compared with those receiving other regimens: 9.8 months (95% CI 8.3, 12.8) for eribulin, 7.2 months (95% CI 5.8, 10.3) for gemcitabine, and 9.1 months (95% CI 6.3, 15.4) for capecitabine [9].

Eribulin is categorized as an anti-tubulin agent with a median PFS of approximately 4 months [3]; however, various non-mitotic effects of eribulin have been reported that could explain the discrepancy between OS and PFS, including vascular remodeling [18, 19], inhibition of the epithelial-mesenchymal transition (EMT) [20], and improvement of the tumor microenvironment [19, 21]. Suppression of transforming growth factor-β1 by eribulin could also have a favorable anti-angiogenic effect, and eribulin therapy leads to remodeling of the microvasculature [18]. Remodeling of abnormal tumor vasculature leads to a more favorable microenvironment that may reduce the aggressiveness of tumors because of the elimination of hypoxic regions. Eribulin therapy may contribute to its clinical benefits [19, 21] by rendering residual tumors less aggressive and less likely to metastasis through an EMT-reversal effect [20]. Furthermore, Kashiwagi et al. reported that eribulin suppressed the expression of EMT and hypoxia markers ABC patient specimens. These results included clinical data on improved survival among patients treated with eribulin, as well as the proposed mechanism underlying this response [22].

In the present study, eribulin resulted in a numerically longer OS from the initiation of first-line chemotherapy compared with conventional chemotherapy; however, eribulin did not demonstrate a statistically significant survival benefit for HER2− ABC patients (869 vs. 744 days, *P* = 0.47, log-rank). A systematic review and meta-analysis of randomized clinical trials for ABC patients showed that a longer duration of first-line chemotherapy was associated with improved OS (HR 0.91; 95% CI: 0.84–0.99, *P* = 0.046) [23]. Thus, OS from the initiation of first-line chemotherapy may be affected by the duration of first-line chemotherapy. In our previous report based on the same database using propensity score matching, eribulin therapy as first-line chemotherapy showed a significantly shorter time to treatment failure (TTF) (HR 1.81; 95% CI: 1.04–3.14, *P* = 0.050) and inferior OS (HR 2.49; 95% CI: 1.38–4.50, *P* = 0.006) compared with paclitaxel plus bevacizumab [10]. In this study, the median TTF and OS for first-line chemotherapy were significantly shorter in the eribulin group than in the non-eribulin group (TTF: 111 days vs. 182 days HR 1.63; 95% CI 1.04–2.56, log-rank *P* = 0.031; OS: 457 days vs. 744 days HR 2.09; 95% CI 1.28–3.40, log-rank *P* = 0.003). This may be one reason why the present study resulted in no statistically significant survival benefit from eribulin therapy in all HER2− ABC patients.

The upfront use of eribulin is not common in most countries where eribulin therapy is subject to reimbursement [1, 2]; thus, we investigated the effect of eribulin on OS and focused on the treatment line of eribulin. There was no difference in median OS from the initiation of first-line chemotherapy in the eribulin group among patients who received eribulin at an early line (first or second) compared with the non-eribulin group (*P* = 0.15, rog-rank). The early-line eribulin group included more patients with recurrent disease (60.5% vs. 81.8%, *P* = 0.001) and more patients with a history of perioperative anthracycline- and/or taxane-based therapy (41.1% vs. 67.0%, P < 0.001) than the non-eribulin group. We could not rule out that this affected the survival benefit resulting from early-line eribulin. On the other hand, our results showed that the median OS from the initiation of first-line chemotherapy was significantly higher in the eribulin group among patients who received eribulin at a later line (third or later) compared with the non-eribulin group (*P* = 0.037, Wilcoxon). Furthermore, patients who had received perioperative anthracycline- and/or taxane-based regimens showed an improved median OS from the initiation of first-line chemotherapy compared with the non-eribulin group (*P* = 0.032, Wilcoxon), In a multivariate analysis, we found that eribulin therapy at a later line (third or later) was an independent predictor of OS from the initiation of first-line chemotherapy (HR 0.39; 95% CI: 0.21–0.70, *P* = 0.002). Our results appear identical to those of prospective [3–6] and retrospective studies [8, 9] that targeted patients with heavily pretreated HER2− ABC and patients who had received anthracycline- and/or taxane-based regimens. However, the fact that patient survival was improved from the initiation of first-line chemotherapy for ABC is a new and important finding.

Biomarkers related to eribulin treatment have been discussed and novel findings of eribulin have been derived not only from laboratory studies [24, 25], but also from the clinic. Miyagawa et al. focused on peripheral immune-related markers, such as the baseline neutrophil-to-lymphocyte ratio (NLR) and showed that NLR was a predictive marker for eribulin therapy [26]. Furthermore, the absolute lymphocyte count (ALC) has been demonstrated to be a predictive factor for eribulin therapy in ABC patients [27–29]. Furthermore, we showed the predictive value of peripheral immune-related markers; such as NLR, ALC, platelet-to-lymphocyte ratio, and lymphocyte-to-monocyte ratio in paclitaxel plus bevacizumab therapy [30], thus, discoveries of novel biomarkers in HER2- ABC patients to maximize the benefit from chemotherapies are warranted.

Several limitations are associated with the present study. This study was retrospective in nature, which may have led to unintended selection bias, so the interpretation and generalization of the results should be considered with care. However, as a strength, our study utilizes relatively large-scale real-world data of patients based on actual clinical practice, which may assist in making judgments consistent with actual clinical practice for the management of HER2− ABC. In addition, we could not rule out that patients who were able to receive eribulin therapy were able to use it because of slowly progressing breast cancer. Further research, especially prospective translational studies, is needed to identify predictors with respect to the response of ABC patients to eribulin therapy.

## Conclusions

While an improvement in OS from the initiation of first-lime chemotherapy for HER2− ABC patients remains challenging, we successfully identified subgroups of HER2− ABC patients who had improved OS from the initiation of first-line chemotherapy that were treated with eribulin therapy. These patients include those who received eribulin therapy at a later line (third or later) and received perioperative anthracycline- and/or taxane-based regimens. To maximize the benefit from eribulin therapy, the discovery of novel predictive factors are needed.

## Data Availability

The datasets used and/or analysed during the current study are available from the corresponding author on reasonable request.

## Abbreviations

ABC: advanced breast cancer
ALC: absolute lymphocyte count
CI: confidence interval
ER: estrogen receptor
EMT: epithelial-mesenchymal transition
HER2−: human epidermal growth factor receptor-2 negative
HR: hazard ratio
NLR: neutrophil-to-lymphocyte ratio
OS: overall survival
PFS: progression-free survival
TPC: treatment of the physician's choice
TTF: time to treatment failure
